# A multivariate data analysis approach for investigating daily statistics of countries affected with COVID-19 pandemic

**DOI:** 10.1016/j.heliyon.2020.e05575

**Published:** 2020-11-24

**Authors:** Ahmed Ramadan, Ahmed Kamel, Alaa Taha, Abdelhamid El-Shabrawy, Noura Anwar Abdel-Fatah

**Affiliations:** a Data Science and Medical Information Department, DataClin Contract Research Organization, Egypt; b Department of Biostatistics and Demography, Faculty of Graduate Studies for Statistical Research, Cairo University, Egypt; c Medical Affairs Department, DataClin Contract Research Organization, Egypt

**Keywords:** Applied mathematics, Virology, COVID-19, Multivariate, PCA, PAM, Visualization, Clustering

## Abstract

**Background:**

To understand the impact and volume of coronavirus (COVID-19) crisis, univariate analysis is tedious for describing the datasets reported daily. However, to capture the full picture and be able to compare situations and consequences for different countries, multivariate analytical models are suggested in order to visualize and compare the situation of different countries more accurately and precisely.

**Aims:**

We aimed to utilize data analysis tools that display the relative positions of data points in fewer dimensions while keeping the variation of the original data set as much as possible, and cluster countries according to their scores on the formed dimensions.

**Methods:**

Principal component analysis (PCA) and Partitioning around medoids (PAM) clustering algorithms were used to analyze data of 56 countries, 82 countries and 91 countries with COVID-19 at three time points, eligible countries included in the analysis are those with total cases of 500 or more with no missing data.

**Results:**

After performing PCA, we generated two scores: Disease Magnitude score that represents total cases, total deaths, total actives cases, and critically ill cases, and Mortality Recovery Ratio score that represents the ratio between total deaths to total recoveries in any given country.

**Conclusion:**

Accurate multivariate analyses can be of great value as they can simplify difficult concepts, explore and communicate findings from health datasets, and support the decision-making process.

## 1 Introduction

On December 31, 2019, the outbreak began in Wuhan, a province in China. Reported cases of “pneumonia of unknown origin” originated from Huanan Seafood Wholesale Market, where some animals like bats, snakes, and rabbits are raised in captivity for consumption by people and are illegally sold. A few days later, the Chinese government confirmed that this outbreak is caused by a novel Coronavirus which was named later by the World Health Organization (WHO), COVID-19 (Bai et al., 2020).

On March 11, 2020, and based on further assessments, WHO Director-General made an announcement that COVID-19 can be characterized as a pandemic (Wu and McGoogan, 2020). By March 16, 2020, the outbreak outside China increased drastically and the number of countries, states, or territories reporting infections to WHO had reached 143 (Huang et al., 2020).

As the situation escalates day by day, there is a growing need for a visualization tool to guide better understanding on the disease pandemic nature (Yoo, 2020). Reported data from the affected countries are important to understand the disease risk and guide different preventive measures. The reports include confirmed cases, confirmed deaths, total recoveries, severe cases, and recovered cases ratio. The data show how countries are promptly working to control the pandemic and trying to preserve the resources to fight the disease spread. They are also sharing practices and strategies needed to ensure that patients are best managed (Dey et al., 2020).

It is very important to consistently record and report epidemiological information for better understanding of disease transmission, geographic spread, risk factors for infection, and different routes of transmission. Also, to provide the baseline for various epidemiological modeling that can guide authorities for optimum planning to minimize the disease burden. This detailed and accurate information is very important to decide where surveillance should be prioritized (Xu et al., 2020).

To capture more clear information effectively, statistical analyses along with data visualization are needed to serve as applications of the powerful models of data science. The role of data scientists now is more important than ever for identifying different trends, patterns, and outliers to help researchers and decision makers to act in a more effective manner towards medical researches and preventive public health measures (Valdiserri and Sullivan, 2018).

Healthcare professionals have acknowledged for so long the importance of conventional disease mapping and geographic information systems (GIS), as some of the most important tools against the fight of an outbreak. The very first disease map that was drawn to visualize the relationship between a disease and its origin was in 1964 on plague outbreak in Italy (Lipton, 2019). Disease maps would be valued and used over the next 25 years aiming to understand and track most of the infectious diseases such as Yellow fever, Cholera, and Influenza (Lyseen et al., 2014).

There are many clinical outcomes reported from different countries affected with COVID-19, these outcomes are likely to have potential correlations with each other. Multivariate analysis is needed to explain interactions among variables present in the dataset, allowing data dimension reduction for better visualization, better hypothesis testing, and explanation between the dataset, so we can have a better understanding to the data reported from affected countries (Clark, 2013; Riley et al., 2017; Williams and Babbie, 1976).

The current study aims to initiatively utilize the widely applied multivariate statistical procedures, PCA and PAM algorithms, for efficient visualization and comparative inference of COVID-19 status in different countries. PCA is commonly used to reduce the number of variables that exist in many datasets, which indeed exhibit multicollinearity that alters the visualization and the application of many statistical techniques and algorithms. Of the featured advantages of PCA, it results into orthogonal components, i.e. uncorrelated/independent factors. On the other hand, PCA may result in illogical or non-interpretable factors when they are formed of non-homogenous set of variables, as it relies on analyzing the correlation matrix of the variables, the results may not make sense in some cases. Hence, a careful evidence-based naming and interpretation of the formed factors should be conducted. Finally, PCA may lead to losing some information since the resulted factors usually explains a percentage of the variability existing in the original dataset. But usually the cumulative percentage of total variance explained is also used as a criterion to judge on the quality of PCA since acceptable results that explains at least 70% of the total variability (Karamizadeh et al., 2013; Lloret et al., 2017; Stewart et al., 2014).

We performed PCA algorithms on five originally reported variables (Total confirmed cases, Active cases, Total deaths, Critically ill cases, and Mortality recovery ratio). We further performed PAM clustering algorithms on the scores of different countries on the reduced dimensions (PC scores), thus we were able to better visualize, categorize and better describe the status of countries affected with COVID-19 pandemic.

## 2 Methods

We captured the available data about Coronavirus statistics from Worldometer website https://www.worldometers.info/coronavirus/ for March 30, April 15, and April 25, 2020. Data were captured on the next day to these specified dates. Countries with COVID-19 total cases less than 500 or countries with missing data were omitted from the analysis to keep good representability of each variable. Number of countries included in the analysis was 56 countries on March 30, 82 countries on April 15, and 91 countries on April 25. Data manipulation and analysis were performed using *R software* (R Core Team, 2019).

We used the following description for each of the variables included; in any given country, total cases refers to total cases confirmed with COVID-19; active cases refers to total number of open cases (mild, serious, or critical); total deaths refers to total deaths with COVID-19; critically ill cases refers to number of serious/critically ill cases; mortality recovery ratio refers to the ratio between total deaths to total recovered patients.

Correlation matrices were visualized using performance analytics package (Peterson et al., 2014). Principal component analysis (PCA) was performed using *FactoMineR* package (Lê et al., 2008). Observations within each variable were converted to Z-scores and subjected to PCA at each time point. The main aim of PCA was to summarize patterns of a relatively large number of observed variables into a smaller number of latent factors that should be able to reflect the underlying processes that caused eventually the correlations among the variables. Mathematically, PCA develops linear combinations of observed variables; each of them is a factor, these factors summarize the pattern of correlations in the observed correlation matrix (Tabachnick and Fidell, 2007). Contributions and correlations of variables with the formed factors were determined at each time point.

We performed cluster analysis using *cluster* and *Factoextra R* packages (Kassambara et al., 2017; Maechler et al., 2013). Partitioning around medoids (PAM) algorithm was utilized to cluster the countries according to their PC-1 and PC-2 scores on the latest time point (April 25). PAM algorithm is a robust alternative to K-means clustering that is less sensitive to noise and outliers (Salgado et al., 2016). Optimum number of clusters was determined according to the highest average silhouette width (Kaufman and Rousseeuw, 1990). We performed successive waves of removal of noise clusters then reassessed the contributions and correlations of variables with the formed dimensions.

We made projection on March 30 model utilizing data of April 15 and April 25. Initial PC scores on March 30 and projected PC scores at the next time points were compared with Friedman ANOVA for the countries whose data were available at the specified three time points. The experimental methodology and design are summarized in a flow chart form (Supplementary material 1).

## 3 Results

**Figure 1:**
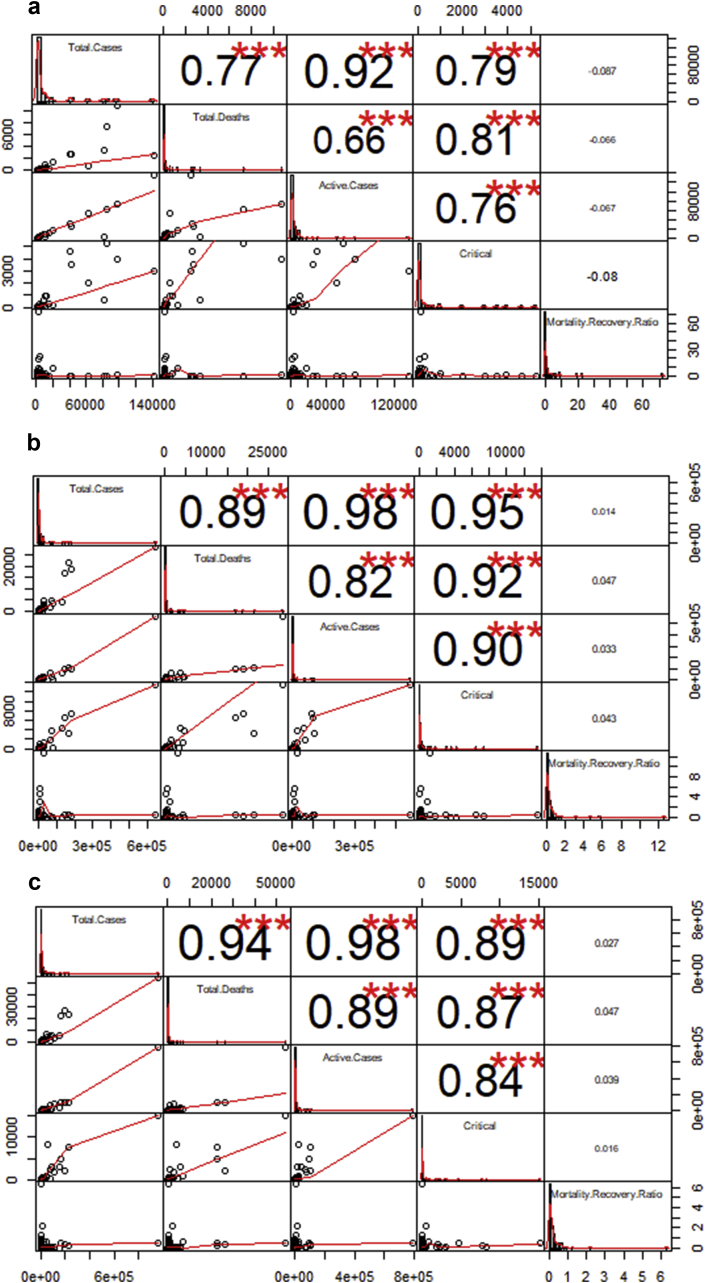
(a) Correlation matrix between the original variables on 30 March 2020. (b) Correlation matrix between the original variables on 15 April 2020. (c) Correlation matrix between the original variables on 25 April 2020.

Descriptive statistics of the original variables at each time point (30 March, 15 April, and 25 April 2020) are presented in (Table 1). The univariate outlier analysis showed the presence of many outliers across all tested variables. However, removal of univariable outliers would have caused a large portion of data to be excluded; so, we made successive waves of noise removal after performing PCA and cluster analysis. Correlation matrices between variables at each time point are presented in (Figure 1). Total cases, total deaths, active cases, and critically ill cases were consistently strongly correlated. On the other hand, mortality recovery ratio had a unique pattern of variance through the tested time points.

**Table 1:** Descriptive analysis for the selected variables.

Time Point	Parameter	Total Cases	Total Deaths	Active Cases	Critical	Mortality Recovery Ratio
30-Mar-2020	Min	515	1	99	1	0.01
	1st Qu	954.8	9.75	866.8	11	0.0775
	Median	1937	26.5	1867.5	38	0.335
	Mean	12789.1	611.55	9416.5	499.1	3.1704
	3rd Qu	7015.8	122	4628.2	170.5	1.325
	Max	142746	10779	135695	5231	72
15-Apr-2020	Min	626	3	61	1	0.01
	1st Qu	1297	30.75	1006	14.25	0.05
	Median	3705	96.5	2764	60.5	0.17
	Mean	23723	1463.67	16143	602.51	0.5683
	3rd Qu	10100	345.75	6625	159.5	0.4175
	Max	644089	28529	566859	13487	12.54
25-Apr-2020	Min	513	4	54	1	0.01
	1st Qu	1444	31.5	842	8.5	0.035
	Median	4361	94	1930	44	0.09
	Mean	29392	1933.9	18455	610.2	0.2722
	3rd Qu	12865	456.5	8108	143	0.245
	Max	960651	54256	788233	15110	6.28

**Figure 2:**
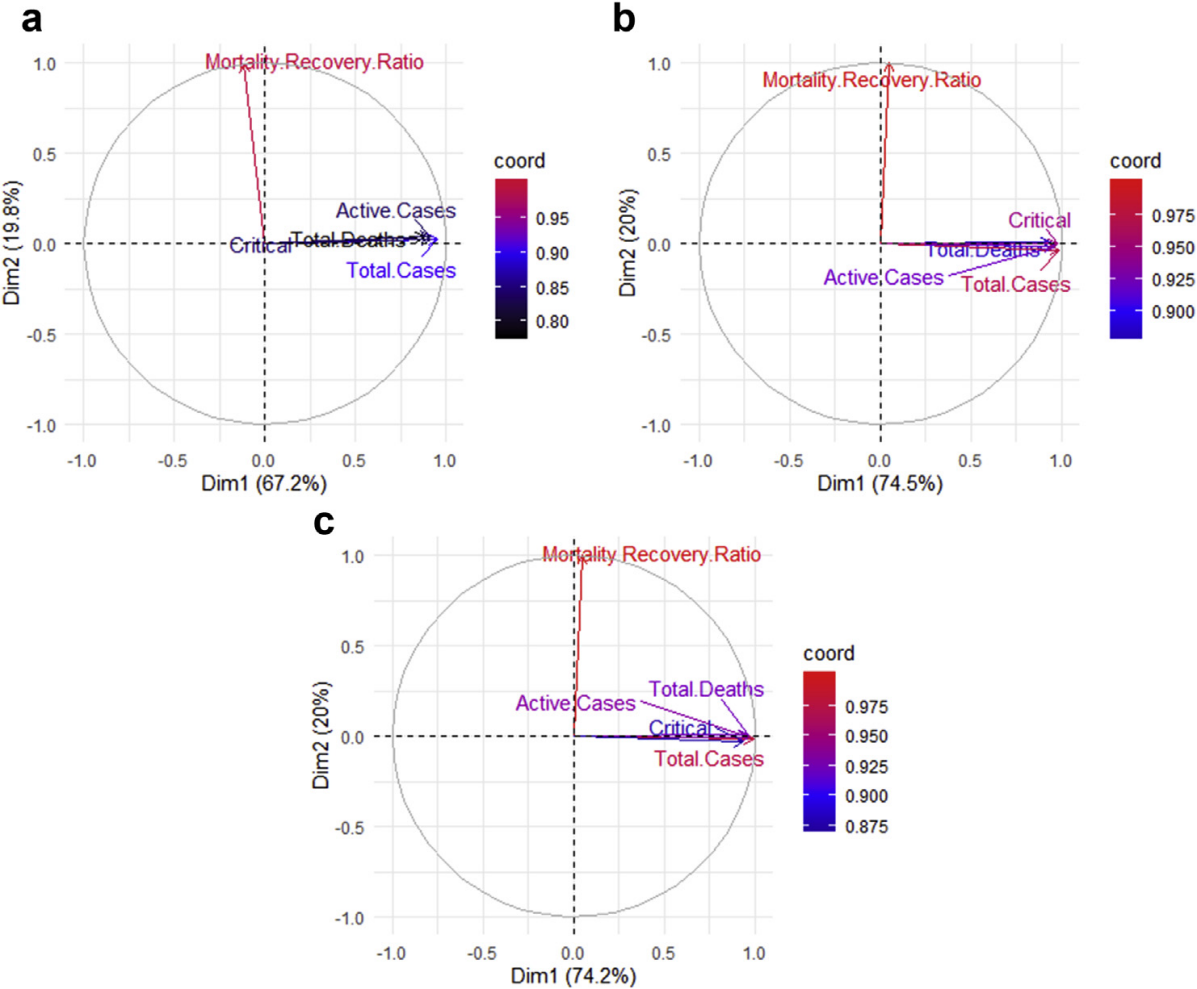
(a) Heat map presenting the correlation between the original variables and the formed factors on 30 March 2020. (b) Heat map presenting the correlation between the original variables and the formed factors on 15 April 2020. (c) Heat map presenting the correlation between the original variables and the formed factors on 25 April 2020.

Upon performing multivariate PCA at each of the three time points, the variables (Total cases, total deaths, active cases, and critically ill cases) were formed into one principal component (PC-1), that we called “Disease Magnitude”, as they had higher loading scores on this formed factor in the three models; while “Mortality-Recovery Ratio” was formed into another principal component (PC-2), as it had higher score on this formed factor. The percentage of contribution of original variables to the formed factors at each time point are presented in (Table 2). The correlation of the original variables with the formed factors are presented in (Figure 2), which presents prefect correlations of the original variables with their relevant formed factors (r > 0.8). The models retained about 87%, 95% and 95% of the total variance within the original variables at each time point, respectively. The loading scores on PC-1 suggested nearly equal contribution of each variable in forming the principal component. Communalities of PC-1 variables (Percentage of explained variance in each variable by the formed principal component) were consistently above 80%, while PC-2 was explaining about 100% of variance of mortality recovery ratio.

**Table 2:** Percentage of contribution to PCs at each time point.

Time Point	Parameter	PC-1	PC-2
30-Mar-2020	Total Cases	26.85608277	0.053066567
	Total Deaths	23.11722557	0.147716968
	Active Cases	24.69648432	0.154690657
	Critical	24.92980116	0.069682407
	Mortality Recovery Ratio	0.400406173	99.5748434
15-Apr-2020	Total Cases	26.15492901	0.124785181
	Total Deaths	23.63840822	0.002791971
	Active Cases	24.68984622	0.026416251
	Critical	25.45451308	0.000735775
	Mortality Recovery Ratio	0.062303474	99.84527082
25-Apr-2020	Total Cases	26.3734431	0.032498244
	Total Deaths	24.89942018	0.002296381
	Active Cases	25.17725371	0.002195602
	Critical	23.49289556	0.091217583
	Mortality Recovery Ratio	0.056987461	99.87179219

The sign of the loading scores on PC-1 was positive in the three models, so the increment in PC-1 scores indicates higher total cases, total deaths, active cases, and critically ill cases. Sign of PC-2 loading score also indicates that increment in PC-2 refers to higher mortality recovery ratio.

**Figure 3:**
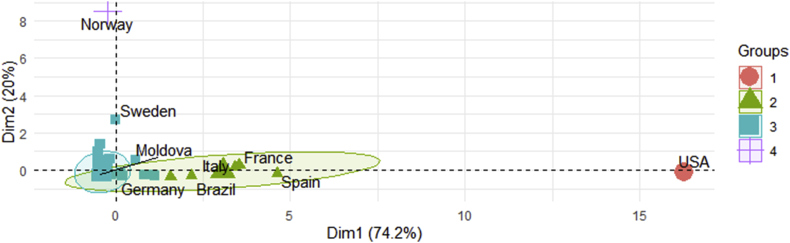
Clustering of countries according to their PC scores on 25 April 2020.

At each time point, each country had two scores for two dimensions, the first score (PC-1 or Disease Magnitude score) simultaneously representing the counts of total cases, total deaths, active cases and critically ill cases, and the other score (PC-2 or Mortality recovery ratio) representing the ratio between total deaths to total recoveries. The two formed variables of PC scores have efficiently stored the information within the original five variables at each time point. The descriptive statistics for both PC scores of countries in the three models are presented in (Table 3). The PC-1 and PC-2 scores of 91 countries on April 25 were subjected to successive waves of cluster analysis utilizing PAM algorithm. Each cluster was represented with one country as a “Medoid”. The medoid country had minimal average dissimilarity with the other members of the cluster and was considered as centroid for each cluster. The medoid was presented by the relevant country scores on PC-1 and PC-2. At the first wave of cluster analysis, the highest average silhouette width suggested that optimum number of clusters was four. Hence, the first wave resulted in four clusters, USA (16.263, -0.113) was solely representing cluster 1, Italy (3.416, 0.171) was the medoid of cluster 2 which contained Spain, Italy, France, Germany, and Brazil. Moldova (-0.452, -0.220) was the medoid of cluster 3 which contained 84 countries of 91 countries in total. Finally, Norway (-0.224, 8.546) was representing cluster 4 (Figure 3).

**Table 3:** Descriptive statistics for PC scores of countries at three time points.

Time Point	Parameter	PC-1	PC-2
30-Mar-2020	Min	-1.0783	-0.35948
	1st Qu	-0.7619	-0.34469
	Median	-0.719	-0.28947
	Mean	0	0
	3rd Qu	-0.4824	-0.07821
	Max	7.17	6.69222
15-Apr-2020	Min	-0.5908	-0.3961
	1st Qu	-0.5725	-0.3184
	Median	-0.523	-0.2522
	Mean	0	0
	3rd Qu	-0.3959	-0.1066
	Max	14.5236	7.4888
25-Apr-2020	Min	-0.5373	-0.36979
	1st Qu	-0.5212	-0.33052
	Median	-0.4711	-0.24926
	Mean	0	0
	3rd Qu	-0.3432	-0.05522
	Max	16.2632	8.54622

Our approach builds on the idea of decomposing the biggest cluster produced in former wave into an optimum number of clusters until we get a meaningful endpoint. To achieve that, first we have to perform a new PCA on the original dataset of this cluster in order to detect any new correlation pattern between the tested variables and the subsequent changes in loading scores on the principal component away from the influence of noise clusters. PCA was reperformed on the original dataset of countries in cluster 3 in the previous model and followed by PAM cluster analysis. The optimum number of clusters in the second clustering step was 2. Iran (7.801, -0.823) was the medoid of cluster 1 which contained Turkey, Iran, Russia, and Belgium; while Finland (-0.618, -0.346) was the medoid of the rest 80 countries.

**Figure 4:**
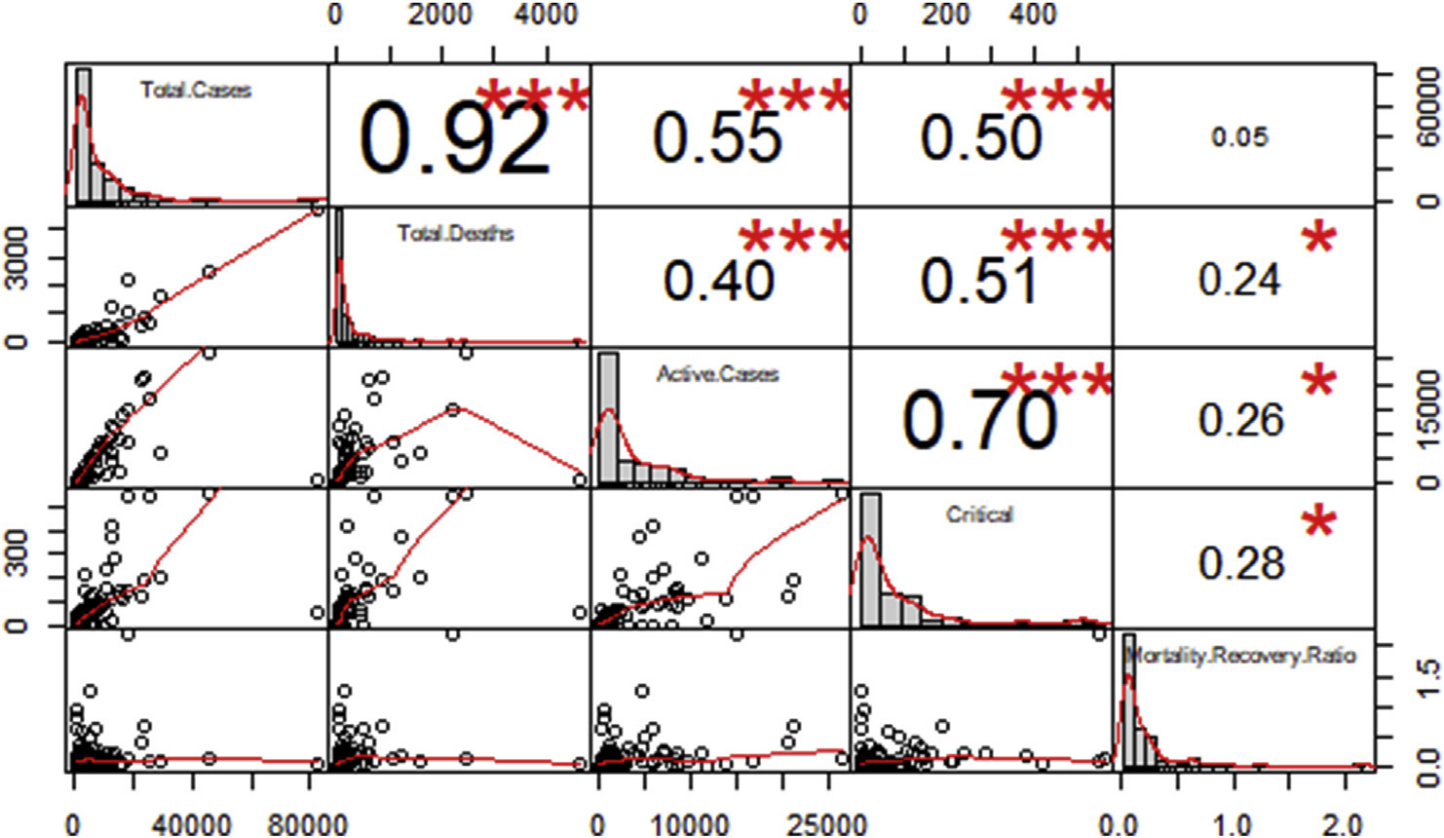
Correlation matrix between the original variables after successive removals of outlier countries.

**Figure 5:**
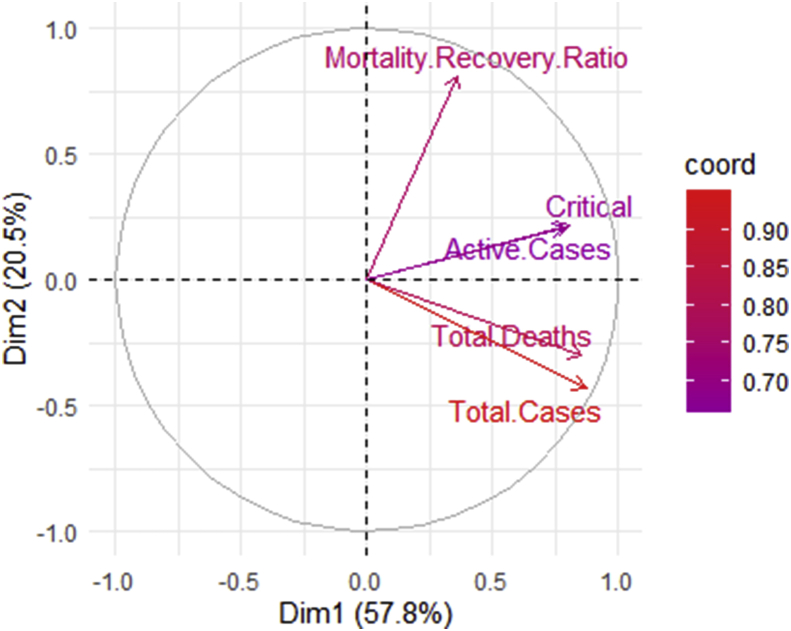
Heat map presenting the correlation between the original variables and the formed factors on 25 April 2020 after successive removals of the outlier countries.

Upon performing the final PCA on the original dataset of the 80 countries in cluster 2 in the previous model, significant weak to moderate correlations between mortality recovery ratio and the rest of variables on PC-1 were observed (Figure 4). Changes were detected accordingly in the correlations between the original variables and the formed factors compared to the model performed initially on 91 countries (Figure 5).

**Figure 6:**
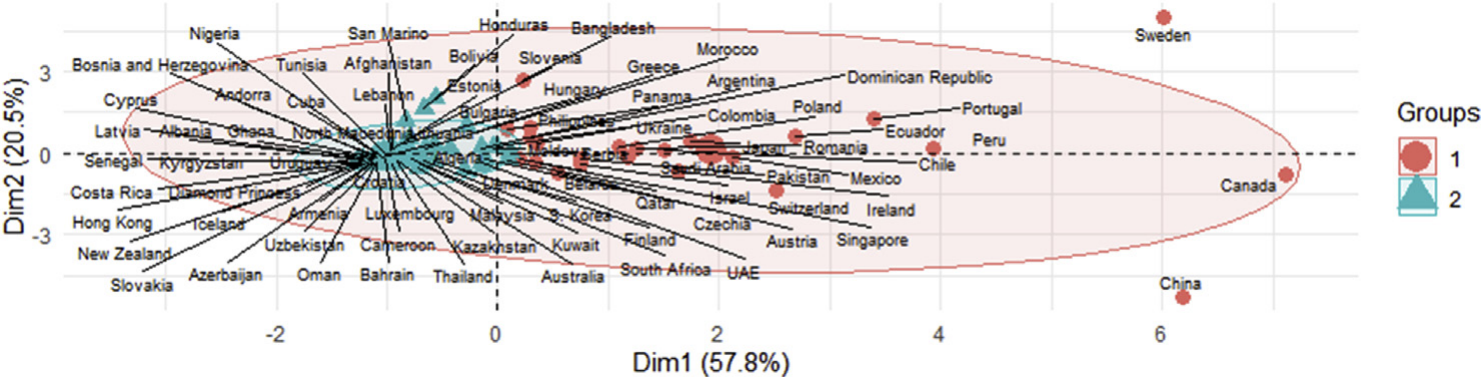
Clustering of countries according to their PC scores on 25 April 2020 after successive removals of the outlier countries.

The 80 countries were further optimally clustered into 2 groups. Romania (1.249, 0.165) was the medoid of the first group which contained 24 countries, and Cameroon (-1.015, -0.184) was the medoid of 56 countries (Figure 6).

We were also concerned with tracking changes in PC scores across the 3 time points. This was vital for detecting significant changes in the variables that contribute to each principal component. We made projection on March 30 model with the data of 15 and April 25. PC-1 and PC-2 scores of March 30's model, projected PC scores of 15 and April 25 were tested by Friedman ANOVA test. PC-1 scores were found to be significantly changed (P = 0.000), while PC-2 scores were insignificantly changed (P = 0.946).

## 4 Discussion

An overwhelming number of studies shed the light on COVID-19 from various dimensions: medical, biological, and epidemiological dimensions, its social correlates and its implication, its impact on economic status worldwide and even on micro-level. A few studies focused on tracking COVID-19 data, for the purpose of summarizing and organizing these data and to find solutions for how this huge amount of data should be visualized and presented into one or two representative graphs. Among the initial descriptive mathematical models for COVID-19 was that introduced by N. E. Huang and F. Qiao. They aimed at tracking the disease course with detecting the efficacy of the local interventions made for disease containment. Despite being robust, it did not provide real-time comment on the disease burden and progression across countries (Huang and Qiao, 2020). Q. Lin et al. developed a conceptual model based on 1918 influenza pandemic modeling framework in London, UK, taking into consideration the governmental actions and individual reactions trying to to forecast the disease behavior patterns of COVID-19 under different scenarios. The model functioned well in forecasting COVID-19 behavior when applied to data from Wuhan, China, but it was built on a unidimensional dependent variable, total confirmed cases (Lin et al., 2020). Dey and colleagues exerted valuable efforts to gather and analyze epidemiological data on COVID-19 outbreak from many open datasets. They utilized visual exploratory data analysis procedures on the available datasets for certain provinces of China and outside China, from 22 January to 16 February 2020. The datasets contained number of confirmed cases, deaths, and recovered cases. They draw heat-maps and heat-bar graphs for china and outside, this was done for each indicator separately and comparisons were done in a univariate manner of analysis (Dey et al., 2020). Another research aimed to develop predictive model for predicting COVID-19 cases, deaths, and recoveries. The researchers utilize SEIR modelling to forecast COVID-19 outbreak inside and outside China based on the daily observations. According to the developed model, they assumed that the outbreak would reach its peak in late May 2020 and would start to drop around early July 2020. They also found that negative sentiments about the virus are more prevailed than positive ones. Positive sentiments were mainly reflected through articles about “collaboration and strength of individuals in facing this epidemic’, while negative articles were related to “uncertainty and poor outcomes of the disease such as deaths” (Binti Hamzah et al., 2020). Another modelling study tried to identify individuals at high risk of severe COVID-19 and how this varies between countries. The identification process was based on individual's age, sex, country-disease prevalence data, multimorbidity fractions, and infection–hospitalization ratios. This study concluded that men are at higher risk compared to women, elder people are at highest risk categories and at the macro-level, the share of the population at highest risk categories in countries with older populations, countries with high prevalence of HIV/AIDS, Chronic kidney disease, Diabetes, Cardiovascular disease, and Chronic respiratory disease (Clark et al., 2020). It is clearly noticeable that all of the previous studies analyzing COVID-19 data items were using univariate analysis techniques in order to forecast future outcomes or relate to any other individual features/variable in a one to one basis. In other words, none of those studies dealt with COVID-19 data items using multivariate analysis techniques.

A real challenge has emerged, which is how to identify the proper time to escalate or deescalate the nationwide intervention measures along the course of the pandemic. A current need for a robust tool incorporating the at-hand variants based on the available data in a one multivariate analysis, our current work presented here is an example of how visual representation can be enhanced using multivariate analysis techniques. The available visual graphs on the websites tracking COVID-19 status utilize the univariate presentation of data, presenting the progression of confirmed cases or deaths as a function of time (CDC, 2020; Worldometer, 2020). Despite being informative in a way, advanced inference for better decision making needs a more advanced methodology to reproduce high dimensional data into less dimensions, which should facilitate description and comparison of countries. Serving that purpose, we developed multivariate models aiming at studying and visualizing the current situation of every affected country by COVID-19 using PCA and cluster analysis. This was in terms of disease burden against mortality/recovery ratio at a certain time point. This will help further inference by governments and non-governmental organizations (NGO's) committed to respond to COVID-19 burden in their countries, to implement priority public health measures to support national plans and interventions.

In the current study, the affected countries had two numerical variables, in which the information within the original five variables are efficiently stored. The PCA algorithms were performed on the calculated Z-scores of the original variables. That is why the averages of the PC scores on the formed dimensions were consistently equal to zero (Table 3). Hence, countries with positive values of disease magnitude score (PC-1 score >0) had relatively higher confirmed cases, deaths, active cases and/or critically ill cases. Similarly, countries with positive values of mortality recovery ratio score had a relatively higher ratio of mortality to recovered cases, while negative values of disease magnitude or mortality recovery ratio scores indicated a relatively controlled status. This can be explained with the PC scores of USA at the first wave of cluster analysis (16.263, -0.113), despite being far in terms of disease magnitude (presented by PC-1 score, 16.263), the mortality recovery ratio was relatively controlled (presented by PC-2 score, -0.113). This is strongly indicating a well-established healthcare system that could absorb the relatively high disease magnitude without increasing the ratio of mortality compared to recovered cases.

On 25 April, the first wave of cluster analysis detected a meaningful number of noise clusters. USA was solely representing cluster 1 with the maximum disease magnitude score, Italy (3.416, 0.171) was the medoid of cluster, having relatively higher disease magnitude score compared to the main cluster 3 (84 countries of 91 countries in total). Norway (-0.224, 8.546) was solely representing cluster 4 by far in terms of high score on mortality recovery ratio (presented on PC-2). Of note, the second cluster whose medoid is Italy represents a group of countries with shared borders between Italy, Germany, France, and Spain, which may partly account for the grouping in one cluster.

Further PCA was performed on data of countries in cluster 3 in the previous model, followed by PAM cluster analysis. The detected changes in the correlations between the tested variables and the subsequent changes in loading scores on the principal component denoted that noise reduction was needed to extract more data overlapped by the noise clusters in the previous PCA. The number of clusters in this step was 2. Iran (7.801, -0.823) was medoid of cluster 1 which contained Turkey, Iran, Russia, and Belgium while Finland (-0.618, -0.346) was medoid of the rest of 80 countries. Again, geographical proximity does appear to contribute to data explanation by our model. The final multivariate analysis for data of the 80 countries in cluster 2 of the previous model showed significant weak to moderate correlations between mortality recovery ratio and rest of variables on PC-1, it also showed a subsequent changes in contributions to each PC; denoting changes compared to the model performed initially on 91 countries. The 80 countries were further optimally clustered into 2 groups. Romania (1.249, 0.165) was medoid of the first group which contained 24 countries, Cameroon (-1.015, -0.184) was medoid of 56 countries. The change in correlations between mortality recovery ratio and variables on PC-1 along with an encountered pattern of signal homogeneity in both PC-1 and PC-2 simultaneously and reciprocally in cluster 1 and cluster 2 in this wave of multivariate analysis revealed that our model has reached a logical outreach point. Each cluster finally represents a disease pattern where PC-1 representing disease magnitude is changing in the same direction of PC-2 representing mortality recovery ratio. This means that successive waves of PCA and cluster analysis were needed to properly group countries with similar disease patterns for better visualization and subsequent data extraction and projection. Moreover, results interpretation in this last step that showed significant weak to moderate correlations between mortality recovery ratio on PC-2 and rest of variables on PC-1. This may indicate that mortality recovery ratio is more influenced by the disease magnitude in the major 80 country cluster. Meaning that health care systems in these countries are beginning to be inadequately accomodative to the increase in disease magnitude, or may mean that these countries need augmentation of their capacity to regain independence of PC-2 from PC-1 and subsequently more disease control.

The methodology of multivariate analysis utilized in this study represents a powerful tool to describe and visualize data at certain time points to study the disease burden in terms of disease magnitude and outcome in each country by terms of readily available data in the light of the dynamic disease attributes. The formed PCs are more convenient and informative upon proper utilization as dependent variables in further predictive regression models. Using this methodology will enable both the scientific and the policy making communities to better organize, analyze, and visualize these growing data.

## 5 Strengths and limitations

The presented multivariate data analysis approach was quite powerful for storing the information within the daily reported COVID-19 statistics in a lower number of dimensions/variables, resulting in better visualization and enhanced comparative inference. However, the variance pattern of the original variables is changing day to day. Hence, the quality and appropriateness of these multivariate procedures should be tested at single day level. The correlation between the original variables should be strong enough for performing dimension reduction procedures.

## 6 Conclusion

Using multivariate analysis techniques, we were able to develop models and simple data visualization tools that can help in interpreting the status of a given country or cluster of countries. COVID-19 daily published statistics were summarized by two scores, disease magnitude score and mortality recovery ratio score, where these reduced dimensions were efficiently able to store the information within the original datasets. Significant correlations detected between both scores in some countries is a warning alarm for saturation of healthcare systems.

## Declarations

### Author contribution statement

Ahmed Ramadan: Conceived and designed the experiments; Analyzed and interpreted the data.

Ahmed Kamel, Alaa Taha: Analyzed and interpreted the data; Wrote the paper.

Abdelhamid El-Shabrawy, Noura Anwar Abdel-Fatah: Analyzed and interpreted the data; Contributed reagents, materials, analysis tools or data; Wrote the paper.

### Funding statement

This research did not receive any specific grant from funding agencies in the public, commercial, or not-for-profit sectors.

### Competing interest statement

The authors declare the following conflict of interests: Ahmed Ramadan; current employee of DataClin CRO.

### Additional information

No additional information is available for this paper.

## Appendix A Supplementary data

Flow chart_ Supplemtary material 1_V2 Flow chart_ Supplemtary material 1_V2
