# Supplementary material for: A multivariate data analysis approach for investigating daily statistics of countries affected with COVID-19 pandemic
Source: Heliyon. 2020 Nov 24;6(11):e05575. doi: 10.1016/j.heliyon.2020.e05575 (PMC7685045; doi:10.1016/j.heliyon.2020.e05575)
Supplement: Flow chart_ Supplemtary material 1_V2 [file mmc1.docx]

| 201 countries with confirmed COVID-19 cases on 30-Mar-2020 | 207 countries with confirmed COVID-19 cases on 15-Apr-2020 | 212 countries with confirmed COVID-19 cases on 25-Apr-2020 |
| --- | --- | --- |
|  |  |  |
| Excluded:  138 countries with less than 500 confirmed cases  7 countries with missing data | Excluded:  116 countries with less than 500 confirmed cases  9 countries with missing data | Excluded:  109 countries with less than 500 confirmed cases  12 countries with missing data |
|  |  |  |
| 56 countries included in the analysis | 82 countries included in the analysis | 91 countries included in the analysis |
|  |  |  |
| PCA | | |
|  |  |  |
| PC-1  Total cases, total deaths, active cases, and critically ill cases were represented by the term **“Disease Magnitude”** |  | PC-2  Ratio between total deaths to total recovered patients was represented by the term **“Mortality-recovery ratio”** |

|  | | 91 countries included in the analysis | |  | |
| --- | --- | --- | --- | --- | --- |
|  | |  | |  | |
|  | | PCA | |  | |
|  | |  | |  | |
|  | | 1^st^ Clustering | |  | |
|  | | | | | |
| Cluster 1  USA | Cluster 2  Italy is medoid of 5 countries | | Cluster 3  Moldova is medoid of 84 countries | | Cluster 4  Norway |
|  |  | |  | |  |
|  |  | | PCA | |  |
|  |  | |  | |  |
|  |  | | 2^nd^ Clustering | |  |
|  | | | | | |
|  | Cluster 1  Iran is medoid of 4 countries | |  | | Cluster 2  Finland is medoid of 80 countries |
|  |  | |  | |  |
|  |  | |  | | PCA |
|  |  | |  | |  |
|  |  | |  | | 3^rd^ Clustering |
|  | | | | | |
|  | Cluster 1  Romania is medoid of 24 countries | |  | | Cluster 2  Cameroon is medoid of 56 countries |
